# Self-reported parkinsonism features in older autistic adults: A descriptive study

**DOI:** 10.1177/13623613211020183

**Published:** 2021-06-21

**Authors:** Hilde M Geurts, Goldie A McQuaid, Sander Begeer, Gregory L Wallace

**Affiliations:** 1 University of Amsterdam, The Netherlands; 2 Leo Kannerhuis (Youz/Parnassiagroup), The Netherlands; 3 George Mason University, USA; 4 Vrije Universiteit Amsterdam, The Netherlands; 5 The George Washington University, USA

**Keywords:** autism, medication, old-age, parkinsonism, self-report

## Abstract

Prior research suggests that autistic adults are at increased risk for developing the core motor components of Parkinson’s disease (i.e. parkinsonism) although knowledge is limited to date. Therefore, we explore: (1) the prevalence of both continuous self-reported motor features typical of parkinsonism and the screen positivity rate for parkinsonism in two samples of older autistic adults without a suspected intellectual disability: the Netherlands-Sample (*N* = 296, 50–81 years; 183 males; 113 females) and the United States of America-Sample (*N* = 209, 50–83 years; 100 males; 109 females), and (2) whether there are differences between those who Screen+ versus Screen− for parkinsonism. Within the Netherlands-Sample only, the age of onset of the motor features was investigated. Self-reported parkinsonism features were prevalent in both samples (the Netherlands-Sample ~17% screened positive, mean age of onset ranging from 35.9 to 50.4 years; the United States of America-Sample ~33% screened positive). Autistic adults (the Netherlands-Sample) screening positive encountered more health problems than those who screened negative. In both samples, autistic adults who screened positive did not differ on current antipsychotic medication use relative to those who screened negative. The next step is to determine whether a positive screen for parkinsonism on this self-report questionnaire also implies that there is a higher risk for idiopathic Parkinson’s disease.

**Lay abstract:**

Autistic adults without a suspected intellectual disability reported several motor features such as having tremors, and stiffness in one’s legs which are considered to be part of a complex of motor features called parkinsonism. This so-called parkinsonism was remarkably prevalent in middle-aged and older autistic adults in two independent studies (Dutch study: 50–81 years, 183 males, 113 females, all adulthood diagnoses; the USA study: 50–83 years, 110 females, 109 males, majority adulthood diagnosis). Parkinsonism can be part of the progressive motor disease—Parkinson’s disease. Therefore, it is important that future studies, including in-person neurological assessment, determine if (and if so, why) autistic adults who report these motor features are at increased risk for developing Parkinson’s disease.

## Introduction

Parkinson’s disease (PD) is a serious chronic neurodegenerative disorder with a significant loss of striatal dopaminergic neurons. PD has a series of motor as well as non-motor features. These motor features are also known as parkinsonism or the hypokinetic-rigid syndrome. Diagnostic criteria for parkinsonism encompass the presence of bradykinesia (slowness in movements) in addition to at least one other motor symptom, such as resting tremors, (cogwheel) rigidity, and postural instability (Postuma et al., 2015). Antipsychotics exposure or vascular damage (Brigo et al., 2014; de Rijk et al., 1997; Rektor et al., 2018) can lead to parkinsonism, but PD is the most common type of parkinsonism. Besides various other medical health conditions (Bishop-Fitzpatrick & Rubenstein, 2019; Fortuna et al., 2016; Rydzewska et al., 2018), neurodegenerative disorders such as PD are associated with autism spectrum disorder (ASD) (e.g. Croen et al., 2015; Hand et al., 2020).

Based on a study relying on health care claims data (Croen et al., 2015), it was shown that PD and its related disorders were 33 times more likely to occur in autistic (predominantly younger) adults (*n* = 1507) than in adults from the general population (*n* = 15,070).^1^ Among autistic adults, the PD prevalence was estimated at 0.93% (*N*_PD_ = 14) (Croen et al., 2015) in a sample which was skewed toward young adulthood. Based on general population studies (e.g. de Rijk et al., 1997), the estimated prevalence in people over 65 years of age is 2.6% for parkinsonism and 1.6% for PD. Given that only 0.4% of autistic participants included in the Croen et al. study were over 65 years of age, the reported percentage of PD in autistic adults is remarkably high. This association of PD and/or parkinsonism with ASD is expected to grow even stronger with increasing age, as these types of neurodegenerative health conditions are more common in old age (de Rijk et al., 1997; Savica et al., 2013). Indeed, in a more recent study (Hand et al., 2020), which also relied on health care claims data, the prevalence of PD was 6.6% in autistic adults solely over 65 years of age (*n* = 4685) versus 1.2% in the non-autistic control group (*n* = 46,850). In these two studies, 19% (Croen et al., 2015) and 44% (Hand et al., 2020), respectively, of the ASD groups were known to have a co-occurring intellectual disability (ID). In this study, we will focus specifically on older autistic adults with an ASD diagnosis without a suspected co-occurring ID to exclude the possibility that the presence of ID partly drives observed high parkinsonism co-occurrence rates.

Investigating PD-related parkinsonism in autistic populations may be challenging. First, there is possible overlap in presentation of motor symptoms. Some motor symptoms that are part of the parkinsonism complex have also been observed in autistic children and teens (e.g. rigidity/stiffness, bradykinesia, coordination difficulties; see Damme et al., 2015; Green et al., 2009; Hutton et al., 2008; Wing & Shah, 2000). Second, prodromal PD-related symptoms (Mantri et al., 2019; Postuma & Berg, 2016) and PD-associated problems (Hoogland et al., 2017; Litvan et al., 2011; Mantri et al., 2019; Muslimovic et al., 2005) are common in many psychiatric patients. Medical and mental health conditions that co-occur with PD—such as sleep disturbances, gastrointestinal problems, mood disorders, and cognitive problems—are also common across the lifespan of autistic adults (e.g. Bishop-Fitzpatrick & Rubenstein, 2019; Croen et al., 2015; Lever & Geurts, 2016; Nylander et al., 2018). Third, parkinsonism can also arise in the context of using antipsychotics. The hallmark motor signs of drug-induced parkinsonism (Blanchet & Kivenko, 2016; Caroff et al., 2011) can be indistinguishable from early stage idiopathic PD but as PD progresses, the conditions become more distinctive. Antipsychotics are commonly prescribed to autistic adults (Houghton et al., 2017). Prescription rates range from 42.8% (Jobski et al., 2017) to 63% (Nylander et al., 2018) of the adult population. Such prescriptions seem to increase with increasing age (Jobski et al., 2017).

Indeed, in older autistic populations (40+ years; based on two independent observational studies—Study 1 *N*_ASD_ = 19; 50–77 years and Study 2 *N*_ASD_ = 37; 40–71 years, both reported in Starkstein et al. (2015)), PD and parkinsonism were found to be even more prevalent (16%–32%) than in the aforementioned Croen et al. (2015) and Hand et al. (2020) studies. Almost two-thirds of the participating autistic adults had a history of antipsychotic medication use. However, Starkstein and colleagues (2015) found no statistically significant difference in parkinsonism co-occurrence rates between those with (31%) and without (20%) such a medication history. The authors argued that medication use, therefore, did not fully drive the observed high prevalence rate, although the study might have been underpowered to actually detect such a difference. Irrespective of the etiology of the elevated prevalence of PD and parkinsonism features in autistic adults during middle and older adulthood, the study of these relatively common motor features merits investigation, given their potential impacts on well-being in autistic adults.

Running large-scale epidemiological studies examining the co-occurrence rates of ASD and PD/parkinsonism is challenging as a face-to-face consult is needed for a thorough neurological examination to determine the actual presence of specific motor symptoms. This is time-consuming and expensive. To circumvent these problems, (self-report) screening tools focusing on the parkinsonism motor complex have been developed to enable the execution of large-scale population studies into PD and parkinsonism (Dahodwala et al., 2012; Fereshtehnejad et al., 2014). Some of these include physical metrics, which are not always feasible in a screening study. Moreover, the majority of existing screening measures are rather long. Therefore, a brief new measure, the Parkinsonism Screening Questionnaire (PSQ; Fereshtehnejad et al., 2014), was developed. The PSQ includes the most differentiating items from commonly used screening questionnaires while still maintaining good psychometric properties.

In this study, we focused on the prevalence of self-reported parkinsonism features using the PSQ (for details, see section “Method”), in two large samples (*n* = 296 in the Netherlands (NL) and *n* = 209 in the United States of America (USA)) of older autistic adults (50–83 years) without suspected ID. To provide context, we present the Screen+ rates and individual item endorsement rates on the PSQ for autistic adults in both ASD samples alongside existing findings of the PD and comparison groups of the original validation study for the PSQ (Fereshtehnejad et al., 2014). We hypothesized that older autistic adults would show elevated parkinsonism rates compared to expected general population base rates.

In addition, we explored whether autistic adults who screened positive versus those who screened negative differed on demographic and health-related factors. Specifically, we expect to find that autistic adults with parkinsonism-related features are older and are more likely to report medical and mental health problems, and cognitive failures compared to those without elevated parkinsonism features as PD is associated with non-motor symptoms as well as the measured motor symptoms. Given the association between antipsychotics exposure and parkinsonism, we explored whether current antipsychotic medications impacted parkinsonism symptoms. Finally, in the NL sample, we examined when, according to the respondents, each motor symptom emerged (i.e. age of onset) as some might argue that the motor symptoms could well be an intrinsic part of ASD. In this case, one would expect that such motor symptoms were already present early on in life.

## Methods

### Participants

This study includes two samples. The first sample was based on data from a series of online questionnaires acquired via the Netherlands Autism Register (NAR, longitudinal cohort from 2013 to current, see www.nederlandsautismeregister.nl/english). A total of 306 Dutch participants over 50 years of age were recruited through the NAR website, the Dutch Autism Society (NVA), and word of mouth advertising. For this study, we focused on participants from the fourth wave of data collection in 2016–2017 as the relevant measures were administered in that wave (see section “Measures” for details). All participants were diagnosed using the *Diagnostic and Statistical Manual of Mental Disorders* (4th ed.; DSM-IV-TR) or *Diagnostic and Statistical Manual of Mental Disorders* (5th ed.; DSM-5) criteria by licensed psychologists or psychiatrists working independently from this study. Only those participants who reported an official clinically diagnosed ASD were included. Thus, we excluded seven individuals who reported that they had not yet received an official clinical diagnosis and three who reported not to have a clinical ASD diagnosis. The resulting total size of was 296 autistic adults aged 50–81 years of age (22.8% >65 years of age). This sample will be referred to as Sample NL. The second sample was from the USA (Sample USA) and was based on data obtained online via the Simons Powering Autism Research and Knowledge (SPARK; The SPARK Consortium, 2018). A total of 211 autistic individuals aged 50–83 years (22.5% >65 years of age) were recruited via SPARK. Two individuals who were self-diagnosed (i.e. did not have a clinical ASD diagnosis given by a professional) were excluded, resulting in a final sample size of 209. Table 1 presents the details on both samples.

**Table 1.** Participant characteristics: descriptive statistics for ASD-NL and ASD-USA samples.

	ASD-NL (*N* = 296)		ASD-USA (*N* = 209)	
Sex				
Female, *n* (%)	113 (38%)		109 (52%)	
Male, *n* (%)	183 (62%)		100 (48%)	
Age				
Mean (SD)	58.4 (5.9)		59.35 (7.15)	
Range	50–81		50–83.3	
Diagnosis				
Autism	27		16	
ASD	53		64	
Asperger’s syndrome	170		120	
PDD-NOS	46		9	
Age of ASD diagnosis	*n* = 289			
Mean (SD)	50.9 (7.0)		44.4 (16.7)	
Range	23.3–75.5		2–82.7	
Race^a^	–		African American	4
			Asian	1
			Caucasian	179
			Native American/Alaska Native	5
			Native Hawaiian or Pacific Islander	1
			More than one race	18
			Other	6
Ethnicity	–		*n* = 206	
			Latinx descent	14
			Not of Latinx descent	189
			Unknown	3
IQ			–	
>130	79			
116–130	127			
86–115	87			
71–85	1			
Unknown	2			
Education^b^	*n* = 282		No high school	1
			Some high school	4
	LowMiddleHigh	10124148	GED diploma	3
			High school graduate	4
			Trade/vocational school	9
			Associate’s degree	22
			Some college	35
			Baccalaureate degree	61
			Graduate/professional degree	70
Annual income	*n* = 279		<US $20,000	55
	<€10,000	9	US $21,000–35,000	38
			US $36,000–50,000	25
			US $51,000–65,000	13
			US $66,000–80,000	16
			US $81,000–100,000	14
			US $101,000–130,000	16
			US $131,000–160,000	8
			>US $160,000	15
			Unknown	5
			Prefer not to answer	4
	€10,000–30,000	88		
	€30,000–50,000	74		
	€50,000–70,0000	31		
	>€70,000	29		
	Unknown	16		
	Prefer not to answer	32		
Living arrangements^c^			*n* = 207	
With parents/family	14		70	
Alone	117		81	
With partner	142		92	
Independent with support	24		–	
Group with support	2		–	
Partner	*n* = 256			
Yes	180		100	
No	76		109	
Medical diagnosis	*n* = 286			
Yes	190		175	
No	92		34	
Unknown	4		–	
If yes, what				
Allergies	61		–	
Asthma	42		–	
Gastrointestinal	71		–	
Diabetes	16		–	
Dermatological (including eczema)	47		–	
Headaches (including migraines)	42		–	
Sleep disorders	123		–	
Other Med	8		–	
Psychiatric diagnosis	*n* = 286			
Yes	115		178	
No	158		31	
Unknown	13		–	
If yes, what^d^				
ADHD	24		68	
Learning disability	8		1	
Mood disorder	65		165	
Anxiety disorder	22		113	
Schizophrenia spectrum disorders	19		3	
Eating disorder	4		0	
Addictive disorder	9		1	
OCD	0		33	
PTSD	0		67	
Other Psy	13		5	
Psychotropic medications	*n* = 250			
Yes	75		110	
No	168		77	
Unknown	7		22	
If yes, what^e^				
Antipsychotic	27		23	
Antidepressant	38		76	
Sedative	7		6	
Stimulant	7		22	
Anticonvulsant	–		36	
Anxiolytic	–		29	
Other PsyTrop	10		–	
AQ-28 total	*n* = 279		*n* = 208	
Mean (SD)	85.2 (11.4)		86.4 (10.8)	
Range	50–110		55–110	
CFQ total				
Mean (SD)	81.6 (15.5)		–	
Range	30–116			

For frequencies, absolute scores are reported.

SD: standard deviation; ASD: autism spectrum disorder; PDD-NOS: pervasive developmental disorder–not otherwise specified; IQ: intelligence quotient; GED: General Educational Development; ADHD: attention deficit hyperactivity disorder; OCD: obsessive-compulsive disorder; PTSD: post-traumatic stress disorder; AQ-28: Autism-Spectrum Quotient–28-item version; CFQ: Cognitive Failures Questionnaire total score; Other Psy: a mixture of psychiatric diagnoses which were mentioned <3 times in both samples such as language problems, tic disorders, behavioral problems ODD/CD, and personality disorders, OCD, hoarding, and PTSD; mood: mood disorders; Other med: a mixture of medical diagnoses such as epilepsy, visual impairment, auditory impairment, and motor problems; Other PsyTrop: medication classes which did not fit into one of the other classes.

a Please note that in the Netherlands, it is not standard to ask about race, only when crucial for a specific hypothesis one is allowed to record race and/or ethnicity. In this case, we felt it is not crucial. However, according to the American definition of races, the participants are mostly Caucasian.

b Education is for the NL sample divided into three groups: low (primary education, lower vocational education, or special needs practical education), middle (preparatory secondary vocational education; higher general secondary education, college, pre-university education), and high (University of Applied Science or University).

c The sum is greater than the number participants as some participants reported more than one type of living arrangement.

d The sum is greater than the number of “yes” responses as some participants reported more than one psychiatric diagnosis.

e The sum is greater than the number of “yes” responses as some participants reported more than one medication class.

### Measures

Please note that the NL and USA samples were recruited for two separate studies which were independently designed; therefore, some differences in measurement approach between the two samples occur.

#### Socio-demographic information and personal characteristics

Information was collected regarding a series of socio-demographic and personal characteristics, through self-report on intellectual level (Sample NL only), highest obtained educational level, gross yearly income, living situation, whether the respondent had a (romantic) partner or not, mental health problems, medical health problems, and current psychotropic medication use (both samples). Because PD can detrimentally impact cognitive functioning, we quantified cognitive difficulties in Sample NL with the *Cognitive Failures Questionnaire* (CFQ) (Broadbent et al., 1982; Merckelbach et al., 1996). The CFQ is considered to be a valid and reliable (Bridger et al., 2013) 25-item self-report questionnaire used to assess the experience of memory errors, and distractibility in everyday situations. Higher CFQ total scores indicate more cognitive failures.

#### Autism characteristics

In order to characterize autistic traits in both samples, the 28-item version of the Autism-Spectrum Quotient (AQ-28) (Baron-Cohen et al., 2001; Hoekstra et al., 2008; Woodbury-Smith et al., 2005) was utilized. Each item is rated on a 4-point scale, and higher AQ-28 total scores indicate more autistic traits. To further validate the self-reported clinical ASD diagnosis in the samples, we examined how many individuals scored above the ASD cutoff of 65 (Hoekstra et al., 2008).

#### Parkinsonism

Parkinsonism features were assessed in both samples using the PSQ (Fereshtehnejad et al., 2014; a Dutch translation was used in the NL Sample). This six-item self-report measure was based on a list of 25 questions, which were originally part of existing parkinsonism and PD screening measures, and created to provide a rapid, cost-effective, valid tool by which to screen for parkinsonism features in population-based settings. The items that optimally differentiated between parkinsonism (both idiopathic PD (*n* = 147) and atypical PD (*n* = 10); *N*_PD_ = 157; mean age = 59.8 years, range = 32–79 years) and an age-matched comparison group (COMP; *N*_COMP_ = 110; mean age = 59.9 years, 40+ years) based on the negative clinical utility index were selected for the PSQ (Fereshtehnejad et al., 2014). The resulting six-item self-report measure inquires about the presence of motoric features, to which respondents may answer “Yes,” “No,” or “I do not know.” The items focused on experiencing stiffness, tremors, troubles with buttoning and dressing, lack of arm swings when walking, the feeling of being stuck to the floor when starting to walk or wanting to turn, and general motor slowing. PSQ questions differ in the time period they cover. The questions regarding stiffness in one’s legs and the experience of tremors ask whether one has experienced this ever in one’s lifetime, while the other four questions refer to one’s current experience. For both the NL and USA sample, the frequency of each item response was reported as well as the total number of “Yes” answers (PSQ total score). Higher scores indicate more parkinsonism features. Particularly important, we report the total screening score, which weights four of the six PSQ items that independently discriminate PD from COMP. Following Fereshtehnejad et al. (2014), we report a weighted screening score. This weighted score is calculated as follows: (2 × stiffness) + (5 × tremors) + (5 × arm swing) + (5 × stuck on floor). For calculating this screening score, “No” is scored as 0 and “Yes” as 1 (potential range = 0–17; scores reported in Fereshtehnejad et al., 2014: PD mean (M) = 12.8, standard deviation (SD) = 4.3; COMP M = 1.1, SD = 2.1) with a score of 7 serving as the cutoff score distinguishing PD from COMP (Fereshtehnejad et al., 2014). Thus, we report two metrics, the PSQ total score and the PSQ screening score.

The reliability of the PSQ was reported to be good (Cronbach’s *α* = 0.88; Spearman’s item-total correlations ranged from 0.74 to 0.81) and the sensitivity and the specificity of the weighted PSQ screening score were, respectively, 92.9% and 93.6% (Fereshtehnejad et al., 2014). Because we were interested in age of onset of these parkinsonism features, an additional question was added to the PSQ for the NL Sample. For each item endorsed, the age of onset of the motor feature was queried. The mean onset ages per item were additional dependent measures for the NL sample.

### Procedure

In Sample NL, all participants took part in a larger online follow-up study of the NAR. They received a wide range of questionnaires (see www.nederlandsautismeregister.nl/english), and for this study, the PSQ and the CFQ were added for those participants over 50 years of age. Similarly, in Sample USA, all participants took part in a broader online study of adult development/aging of 40+-year-old autistic adults recruited via SPARK, and were compensated US $25 for their time. However, for comparability between samples, only individuals 50+ years of age from Sample USA were included for this study’s analyses. Both studies were approved by their local institutional review boards (NL: E1321MW and VCWE-2020-041; USA: NCR191497) and followed procedures in accordance with the Declaration of Helsinki. Accordingly, all participants from both studies provided informed consent. The studies were not designed or carried out with involvement from the autistic community, but all questionnaires included in the Sample NL are administered via the NAR for which autistic community members need to check and approve all questionnaires before they are included in this register.

### Statistical analyses

Analyses were performed using SPSS version 24 (IBM Corp., 2016) and JASP version 0.9 (JASP Team, 2018) for Sample NL and SPSS version 26 (IBM Corp., 2019) and JASP version 0.13 (JASP Team, 2020) for Sample USA. In order to describe the samples, mean values and standard deviations (for continuous measures) and frequencies (for categorical measures) are reported. Moreover, within each sample, the number and percentage of participants who report PD-related motor symptoms and who score above the clinical cutoff on the PSQ were calculated. Next, each of the ASD samples was split into two groups (Screen+ and Screen−) in order to explore differences between those that surpass the threshold (i.e. obtain a weighted score of 7+ on the PSQ) and those who Screen− for the presence of self-reported parkinsonism features. For the continuous measures (age, age at ASD diagnosis, and AQ-28 in both samples, and CFQ total scores in Sample NL only), independent samples *t*-tests were conducted. For the four categorical measures (sex (male/female), medical (yes/no), mental health problems (yes/no), and psychotropic medication use (yes/no)), Chi-square analyses were implemented. In order to correct for multiple comparisons, *α* was set at 0.006 (0.05/8) across all eight analyses in each of the samples.

In addition to conventional analyses, we performed the Bayesian analyses (JASP Team, 2018, 2020; Kelter, 2020) to assess the strength of evidence for the group comparison findings. Bayesian hypothesis testing quantifies the extent to which the data support an alternative hypothesis H_1_ against the null hypothesis H_0_, as expressed by the Bayes factor (Rouder et al., 2009), BF_10_. A Bayes factor of 1 indicates no evidence for the alternative hypothesis over the null hypothesis (i.e. no difference), 1–3 anecdotal, 3–10 moderate, 10–30 strong, 30–100 very strong, and >100 extreme evidence for the alternative hypothesis (Wagenmakers et al., 2011). The prior was set, following the JASP standard, at 0.05 for the fixed effects.

## Results

### Sample characteristics

#### Sample NL

Table 1 presents the details regarding the Dutch participants’ socio-demographic and personal characteristics. In line with the self-reported clinical diagnosis of ASD, the mean AQ-28 score (85.2) was consistent with scores reported in previous studies of autistic adults (87.8–91.5) (Hoekstra et al., 2008). In addition, nearly all (94.3%) participants scored above the ASD cutoff of the AQ-28 of 65. There were only four participants who reported having received a motor problem diagnosis in the past. However, most of the autistic adults reported other co-occurring medical (67%) and/or mental health (42.1%) problems for which in some instances psychotropic medication (26.9%) was prescribed.

#### Sample USA

As above, demographic details of the sample from the USA can be found in Table 1. The mean AQ-28 score (86.4) was consistent with findings from Sample NL (85.2) and prior studies (87.8–91.5) (Hoekstra et al., 2008), and nearly all participants scored above the AQ-28 cutoff of 65 (96.6%). Like in Sample NL, most of the autistic adults reported co-occurring medical (83.7%) and/or mental health (85.2%) problems, and accordingly, many were taking a variety of psychotropic medications (58.8%).

### Prevalence of self-reported parkinsonism

**Figure 1.**
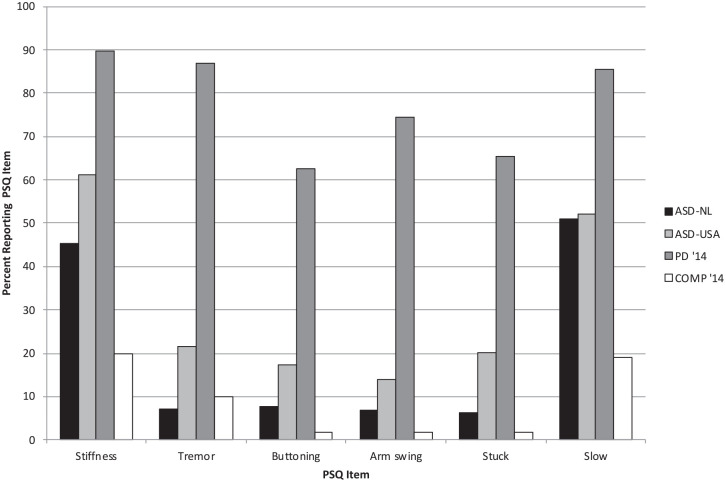
Percentage of ASD-NL (Sample 1) and ASD-USA (Sample 2) participants endorsing each PSQ item. For comparison, the percentage of PD participants (PD’14) and non-PD comparison group participants (COMP’14) endorsing these items (reported in Fereshtehnejad et al., 2014) is also presented.

Figure 1 and Table 2 present a detailed overview of the parkinsonism features results for both samples (for more details, see the Supporting Information).

**Table 2.** PSQ scores of autistic adults in the ASD-NL and ASD-USA samples.

Symptom	ASD-NL (*N* = 296)	ASD-USA (*N* = 209)
Stiffness and rigidity, *n* (%)	134 (45.3)	128 (61.2)
If yes, mean age (SD); range	46.1 (16.3); 1–70	–
If yes, % symptom <18 years	10.8%	–
Tremor and shaking, *n* (%)	21 (7.1)	45 (21.5)
If yes, mean age (SD); range	35.9 (21.8); 1–63	–
If yes, % symptom <18 years	28.6%	–
Troublesome buttoning, *n* (%)	23 (7.8)	36 (17.3)^a^
If yes, mean age (SD); range	36.6 (20.2); 2–62	–
If yes, % symptom <18 years	21.7%	–
Troublesome arm swing, *n* (%)	20 (6.8)	29 (13.9)^a^
If yes, mean age (SD); range	40.2 (19.6); 4–65	–
If yes, % symptom <18 years	21.1%	–
Feet stuck to floor, *n* (%)	19 (6.4)	42 (20.1)
If yes, mean age (SD); range	40.0 (18.8); 8–68	–
If yes, % symptom <18 years	15.8%	–
Motor slowing, *n* (%)	151 (51.0)	109 (52.2)
If yes, mean age (SD); range	50.4 (13.3); 1–75	–
If yes, % symptom <18 years	4.7%	–
PSQ total score, mean (SD)	1.2 (1.2)	1.9 (1.5)
Range	0–6	0–6
PSQ screening score, mean (SD)	1.9 (2.9)	4 (4.2)
Range	0–17	0–17

PSQ: Parkinsonism Screening Questionnaire; ASD: autism spectrum disorder; SD: standard deviation.

The “I do not know” answers ranged from 0.3% (troublesome buttoning) to 7.1 (troublesome arm swing) in the ASD-NL sample, and from 0.96% (troublesome buttoning) to 24.5% (troublesome arm swing) in the ASD-USA sample.

a ASD-USA, *n*= 208.

A total of 64.5% of participants gave an affirmative answer to at least one PSQ item and 16.9% (*N* = 50) scored above the PSQ screening cutoff, compared to 81.3% and 33% (*N* = 69) in Sample USA, respectively. The mean PSQ total score was 1.2 for Sample NL and 1.9 for Sample USA, both of which are in between the PD (4.6) and COMP (0.6) scores as reported in the Fereshtehnejad and colleagues (2014) study. Similarly, the PSQ screening scores of 1.9 for Sample NL and 4.0 for Sample USA are higher than those found in the COMP group (1.1), but still well below the score from the PD group (12.8) in the Fereshtehnejad and colleagues (2014) study. In Figure 1, the percentages of autistic adults from both samples who reported motor symptom are put alongside the percentages for the PD and COMP groups in the original Fereshtehnejad and colleagues (2014) study. Visual inspection reveals that item endorsements of stiffness and general motor slowing in the ASD group were particularly divergent from those reported in the COMP group, but the percentages remain lower than those found in the PD group.

### Age of onset

In Sample NL, the mean age of self-reported emergence (see Table 2) of parkinsonism motor symptoms ranged from 35.9 (tremors) to 50.4 years (general motor slowing), but there was a large range of reported ages of onset (1–75 years). For a minority of the participants, these motor symptoms emerged in childhood (i.e. before 18 years of age), ranging from only 4.7% (general motor slowing) to 28.6% (tremors and shaking).

### Comparisons of parkinsonism Screen+ and Screen− groups

#### Sample NL

When comparing autistic adults who scored above the PSQ cutoff score (Screen+ group *n* = 50) with those who scored below this cutoff (Screen− group *n* = 246), separate *t*-tests revealed that a difference emerged only for the CFQ total score (see Table 3). In contrast to the hypotheses, the Screen+ group reported fewer cognitive failures as compared to the Screen− group. In line with our hypotheses, the Screen+ group self-reported more medical and mental health problems than the Screen− group. These findings were corroborated by the Bayes factors as these indicate that there is strong to extreme evidence in favor of observing an actual difference. The Screen+ and Screen− groups did not differ significantly on any of the other categorical measures. As specifically antipsychotics exposure is associated with parkinsonism features, we explored whether current antipsychotic medication use differed between the Screen+ and Screen− groups. Again, no significant differences emerged (*χ*^2^(1) = 0.60, *p* = 0.44; BF_10_ = 0.027; BF_01_ = 37.04).

**Table 3.** Group comparisons between those who do (Screen+) and do not (Screen−) above the PSQ cutoff score.

	Sample	Group		Statistics	BF_10_	BF_01_
		Screen−	Screen+			
		M (SD; range)	M (SD; range)			
Age	NL	58.4 (6.1; 50.0–80.7)	58.5 (4.9; 50.6–70.5)	*t*(294) = −0.09, *p* = 0.93	0.17	5.94
	USA	59.2 (6.9; 50.0–83.3)	59.6 (7.6; 50.0–78.2)	*t*(207) = −0.31, *p* = 0.76	0.17	6.00
Age of dx	NL	51.0 (6.9; 30.7–75.5)	50.4 (7.6; 23.2–65.9)	*t*(287) = 0.61, *p* = 0.55	0.20	4.92
	USA	46.2 (15.5; 2.0–82.7)	40.8 (18.4; 2.0–70.0)	*t*(207) = 2.07, *p* = 0.04	1.48	0.67
AQ-28 total	NL	85.1 (11.3; 50–110)	85.8 (12.4; 56–110)	*t*(277) = −0.35, *p* = 0.72	0.19	5.39
	USA	86.1 (10.5; 55–108)	87.1 (11.5; 55–110)	*t*(206) = −0.63, *p* = 0.53	0.19	5.20
CFQ total	NL	83.8 (14.5; 37–116)	70.4 (15.7; 30–104)	*t(*294) = 5.89, *p* < 0.001*	92,4942	0.00
Sex (M/F)	NL	155/91	28/22	*χ*²(1) = 0.87, *p* = 0.35	0.17	5.88
	USA	76/64	24/45	*χ*²(1) = 7.05, *p* = 0.008*	5.45	0.18
Medical dx^a^	NL	147/86/3	43/6/1	*χ*²(2) = 12.67, *p* = 0.002*	27.81	0.04
	USA	118/22	63/6	*χ*²(1) = 1.96, *p* = 0.16	0.59	1.70
Mental health dx^a^	NL	87/141/8	28/17/2	*χ*²(2) = 11.31, *p* = 0.003*	15.74	0.06
	USA	116/24	62/7	*χ*²(1) = 1.79, *p* = 0.18	0.52	1.92
Psychotropic medications^b^	NL	62/139/6	13/29/1	*χ*²(2) = 0.43, *p* = 0.98	0.04	25.0
	USA	73/52/0	37/25/3	*χ*²(2) = 5.89, *p* = 0.05	1.15	0.87

PSQ: Parkinsonism Screening Questionnaire; BF: Bayes factor (note that evidence for the null hypothesis as compared to the alternative hypothesis (BF_01_) is 1/BF_10_); SD: standard deviation; dx: diagnosis; AQ-28: Autism-Spectrum Quotient–28-item version; CFQ: Cognitive Failures Questionnaire.

*n* differs for some dependent measures, see Table 1.

a Three answer options in Sample 1: yes/no/unknown and two answer options in Sample 2: yes/no.

b Three answer options: yes/no/unknown.

* Findings are considered statistically significant when *p* < 0.006.

#### Sample USA

There was a greater proportion of females in the Screen+ (*n* = 69) than in the Screen− group (*n* = 140). Yet, this apparent difference did not meet the threshold for multiple comparisons, nor did any of the other comparisons. The Bayes factors were in line with the aforementioned lack of statistical differences. Like in Sample NL, there were no differences with respect to current antipsychotic medication use (*χ*^2^(1) = 0.42, *p* = 0.52; BF_10_ = 0.32; BF_01_ = 3.10).

## Discussion

For the first time, we demonstrate that self-reported parkinsonism features among middle and old age autistic adults without a suspected ID are markedly elevated (~17%–33%) compared to general population expectations ≈2.6% (de Rijk et al., 1997; Fereshtehnejad et al., 2014). Adding to the robustness of these findings, we replicate findings of elevated parkinsonism across two large independent samples from two different countries (NL and USA). The prevalence of PD rates in the two ASD samples is much higher than reported PD rates in health care claims data of autistic adults across predominantly younger (0.93%; Croen et al., 2015) and older adulthood (6.6% Hand et al., 2020). However, the rates of parkinsonism features reported here are comparable to those found among 40+-year-old autistic adults who were examined using a gold standard PD assessment instrument, the Unified Parkinson Disease Rating Scale (16%–32%; Starkstein et al., 2015).

The most common self-reported motor features across both samples were rigidity, stiffness, and slowness. These parkinsonism features might, on the surface, appear to fit with previously described motor behaviors (i.e. rigidity and bradykinesia) found among some autistic children (Damme et al., 2015; Green et al., 2009; Hutton et al., 2008; Wing & Shah, 2000). These features could thus represent pre-existing motor difficulties among some of these autistic adults rather than being a sign of PD-related parkinsonism. However, the rates at which autistic adults in both samples report these behaviors are quite high, and certainly much higher than the comparison group from the original PSQ study. Furthermore, the motoric features that best discriminated PD from the comparison group—getting “stuck” and arm swing differences (in both samples), and tremors (in the USA sample)—are elevated among autistic adults, suggestive of not simply general motor difficulties, but difficulties that overlap specifically with parkinsonism features.

Furthermore, and providing potential evidence that these endorsed motoric features are not mere pre-existing difficulties, the majority of the autistic adults in Sample NL indicated that the queried motor features were not present in childhood, but rather emerged only later, in adulthood. One possible explanation for the high prevalence of parkinsonism in older autistic adults is exposure to psychotropic medications, and, in particular, antipsychotics exposure. Importantly, however, there were no differences in psychotropic medication, and in particular antipsychotics, between those who screened positive versus negative for parkinsonism. In addition, it is critical to point out that drug-induced parkinsonism that fails to remit after the relevant drug is withdrawn, and that in fact progresses, is related to the “unmasking” of a susceptibility to or the exacerbation of what was previously a subclinical progressive case of idiopathic PD (Brigo et al., 2014).

In addition to hypothesizing that a large proportion of autistic adults would Screen+ for parkinsonism features, we expected that those who screened positive would more often experience co-occurring medical and mental health conditions compared to those who screened negative. There is mixed evidence here. While there were differences observed in Sample NL, Sample USA did not demonstrate differences, likely due to the preponderance of co-occurring medical and psychiatric conditions in this latter sample. In addition, given that PD detrimentally impacts cognitive functioning, we expected that elevated parkinsonism features would be associated with more cognitive failures; however, that largely was not the case here. In contrast, autistic people in Sample NL who screened positive for parkinsonism reported fewer cognitive failures than those screening negative. A lack of difference could have followed from the fact that autistic adults often already report a high number of cognitive failures, but this cannot explain why we observed a pattern opposite to what we expected. If this finding is replicated, it might be a hint at the possibility that parkinsonism observed in autistic adults is not the same as parkinsonism observed in people with idiopathic PD.

Notably, the percentage of autistic adults who scored above the clinical cutoff (i.e. Screen+ for parkinsonism on the self-report questionnaire) differs considerably between the two samples. This might be due to the differences between the two samples. For example, in the NL sample, a larger proportion received their ASD diagnosis in adulthood, less often had a co-occurring psychiatric diagnosis, and reported less use of psychotropic drugs. Each of these factors could be relevant in explaining the difference in the prevalence of parkinsonism between the two samples. While the two samples are comparable regarding self-reported autistic traits, being autistic might have different consequences/outcomes in the USA as compared to the Netherlands. Whether this is related to the social care and health care system differences, differences in acceptance, or other differences are beyond the scope of this article. However, one possibility and likely the most parsimonious explanation for the difference in positive screens between the NL and USA sample could be the difference in sex ratio. In the USA sample, more females are included, and in the USA sample, relatively more females than males screened positive for parkinsonism. This is in line with the recent observation that being female and autistic increased the risk of developing health conditions (Rydzewska et al., 2018).

In addition to clear strengths, such as the uniquely large samples of older participants with an ASD diagnosis and the replication of findings in two independent samples, this study has limitations that need to be considered when interpreting findings. First, no standardized ASD assessment tools were used to confirm the reported clinical diagnoses. However, participants were excluded when they self-reported being autistic, but could not provide details regarding the official clinical diagnosis. Moreover, nearly every participant in both samples scored above cutoff on the AQ-28. Second, the PSQ is a new instrument and the findings of the original study are, to our knowledge, not yet replicated. While reliability and validity data for the self-report PSQ are good (Fereshtehnejad et al., 2014), the gold standard approach to evaluation of parkinsonism relies on expert neurological observations of these key motor behaviors. Third, in Sample NL, we also relied on self-reported age of onset of each of the endorsed PSQ features. It could be questioned whether participants can provide reliable and valid retrospective assessment of the onset of these features. A longitudinal cohort, which is not self-selected, in which motor symptoms in childhood are directly measured, would be a needed future research avenue to determine the age of onset of these features. Finally, reported current use of antipsychotics was not very common in both samples, and it was not assessed across the lifespan. While no difference in antipsychotic usage between autistic participants screening positive versus those screening negative for parkinsonism was observed in either sample, it is possible that in a sample in which antipsychotics are more commonly prescribed, or in which antipsychotics have been chronically prescribed, a relationship with parkinsonism motor behaviors might be present.

In summary, keeping in mind this study’s strengths and caveats, the key conclusion of this study is that self-reported parkinsonism features are prevalent in middle and older age autistic adults without a suspected ID. Whether the markedly elevated parkinsonism features among middle and older age autistic adults reflect an idiopathic parkinsonism motor complex occurring in a subset of older autistic adults, or are associated with motoric difficulties emerging earlier in development in autistic individuals, the presence of these features warrants further investigation. Moreover, given the scarcity of existing knowledge of aging in ASD, including the prevalence of disorders associated with aging, such as PD, documenting aging-related syndromes among older autistic adults is critical to understanding well-being and outcomes in autistic persons. Thus, studies that can use a tool such as the PSQ to quickly and cost-effectively screen autistic individuals who can subsequently be brought in for more time-intensive, face-to-face neurological assessments are needed. In addition, longitudinal studies that can determine the age of onset of these motoric features and whether they progress to a PD diagnosis or other parkinsonism syndrome are needed.

## Notes

1. Please note that in this article, we use identity-first (e.g. “autistic adults”) rather than person-first (e.g. “adults with autism”) language when referring to persons, but when referring to a sample, we use the phrase ASD sample/group. While a recent study showed identity-first language was preferred by the majority of the autistic adult population (e.g. Kenny et al., 2016), we do acknowledge that there is an ongoing discussion about this terminology and that some stakeholder groups and individuals prefer person-first language and that this preference differs across countries.
